# A high-quality chromosome-level genome assembly of the Chinese medaka *Oryzias sinensis*

**DOI:** 10.1038/s41597-024-03173-8

**Published:** 2024-03-28

**Authors:** Zhongdian Dong, Jiangman Wang, Guozhu Chen, Yusong Guo, Na Zhao, Zhongduo Wang, Bo Zhang

**Affiliations:** 1 Key Laboratory of Aquaculture in the South China Sea for Aquatic Economic Animals of Guangdong Higher Education Institutes, College of Fishery, Guangdong Ocean University, Zhanjiang, 524088 China; 2 Guangdong Provincial Key Laboratory of Aquatic Animal Disease Control and Healthy Culture, College of Fishery, Guangdong Ocean University, Zhanjiang, 524088 China; 3 Qingdao Marine Management Support Center, Qingdao, Shandong China; 4 National Plateau Wetland Research Center, College of Wetlands, Southwest Forestry University, Kunming, 650224 China; 5 Southern Marine Science and Engineering Guangdong Laboratory-Zhanjiang, Zhanjiang, 524000 China

**Keywords:** Ichthyology, Animal breeding, Genome

## Abstract

*Oryzias sinensis*, also known as Chinese medaka or Chinese ricefish, is a commonly used animal model for aquatic environmental assessment in the wild as well as gene function validation or toxicology research in the lab. Here, a high-quality chromosome-level genome assembly of *O. sinensis* was generated using single-tube long fragment read (stLFR) reads, Nanopore long-reads, and Hi-C sequencing data. The genome is 796.58 Mb, and a total of 712.17 Mb of the assembled sequences were anchored to 23 pseudo-chromosomes. A final set of 22,461 genes were annotated, with 98.67% being functionally annotated. The Benchmarking Universal Single-Copy Orthologs (BUSCO) benchmark of genome assembly and gene annotation reached 95.1% (93.3% single-copy) and 94.6% (91.7% single-copy), respectively. Furthermore, we also use ATAC-seq to uncover chromosome transposase-accessibility as well as related genome area function enrichment for *Oryzias sinensis*. This study offers a new improved foundation for future genomics research in Chinese medaka.

## Background & Summary

Chinese medaka, or *Oryzias sinensis*, is a teleost fish closely related to Japanese medaka (*Oryzias latipes*), which has been used as a model organism in many genomic studies as well as in aquatic toxicology research^1^. Both fish belong to the family Adrianichthyidae, commonly referred to as the Medaka family. Similar to its relative, Chinese medaka is also attracting the attention of scientists due to its small size and short generation interval^2^. There are several differences between these two fishes, including their vertebrae and pectoral fin strip; most importantly, Chinese medaka is mainly distributed in freshwater while Japanese medaka can adapt to a certain level of salinity. Because of this, Chinese medaka could be more suitable for freshwater quality evaluation.

Although the Chinese medaka genome has been released^3^, the completeness and genome annotations still need to be further improved. The reported genome was only released at the scaffold level with alignments to the chromosomes of Japanese medaka. Several phylogenetic analyses and taxonomic revisions for medaka have already found that the Chinese medaka is different from its Japanese relative in chromosome constitution; the diploid chromosome number is 46 in *O. sinensis*, and 48 in *O. latipes*^4,5^. Therefore, a high-quality reference genome for *O. sinensis* is increasingly important to support future research.

In the present study, an improved high-quality chromosome-level genome assembly of *O. sinensis* was generated using single-tube long fragment read (stLFR) reads, Nanopore long-reads, and the Hi-C sequencing data. The genome size was 796.58 Mb with a scaffold N50 length of 30.38 Mb (Table 1). A total of 712.17 Mb (89.34%) of assembled sequences were anchored to 23 pseudo-chromosomes, with lengths ranging from 58.48 Mb to 18.87 Mb (Table 2). Based on this improved genome assembly, repeat elements and gene structure annotations were conducted combining *de novo* prediction, homolog-based alignment, and transcriptome-assisted methods. Benchmarking Universal Single-Copy Orthologs (BUSCO) evaluation result showed that the final assembly was benchmarked at 95.1% and the annotation reached 94.6% (Table 3).

**Table 1:** Genome assembly statistics of Chinese medaka *Oryzias sinensis*.

Statistical level:	New chromosome-level genome			Published genome (GCA_008586565.1)	
	chromosome	scaffold	contig	scaffold	contig
**Total number (>):**	23	17,780	24,324	68,189	98,653
**Total length of (bp):**	712,173,063	797,156,861	765,499,752	813,986,518	734,065,487
**N50 Length (bp):**	31,744,625	30,386,322	373,585	991,358	28,180
**N90 Length (bp):**	24,137,528	36,705	13,816	2,913	2,405
**Maximum length (bp):**	58,480,650	58,480,650	2,853,130	7,585,817	287,113
**Minimum length (bp):**	18,868,712	714	48	747	1
**GC content is (%):**	40.69			40.45

**Table 2:** Chromosome length distribution of assembled genome.

Chromosome ID	Length (bp)	Percentage (%)
1	58,480,650	7.34
2	39,505,166	4.96
3	39,054,318	4.9
4	33,217,277	4.17
5	32,964,224	4.14
6	32,856,377	4.12
7	32,552,571	4.08
8	32,162,499	4.03
9	31,899,507	4
10	31,744,625	3.98
11	31,538,233	3.96
12	30,386,322	3.81
13	29,554,195	3.71
14	29,246,493	3.67
15	28,794,247	3.61
16	28,705,415	3.6
17	27,734,809	3.48
18	27,467,262	3.45
19	24,846,853	3.12
20	24,137,528	3.03
21	23,984,531	3.01
22	22,471,249	2.82
23	18,868,712	2.37
Unplaced*	84,983,798	10.66

*Unplaced referring the sequences which did not mount to known chromosome.

**Table 3:** Genome and Annotation BUSCO evaluation result.

	Genome		Annotation	
	Gene numbers	Percentage	Gene numbers	Percentage
Complete BUSCOs	4,356	95.1	4,335	94.6
Complete Single-Copy BUSCOs	4,275	93.3	4,202	91.7
Complete Duplicated BUSCOs	81	1.8	133	2.9
Fragmented BUSCOs	123	2.7	153	3.3
Missing BUSCOs	105	2.2	96	2.1
Total BUSCO groups searched	4,584	100	4,584	100

In addition, we conducted ATAC-seq try to find out the chromosome accessibility of *O. sinensis*. After we got the peak results we also performed function enrichment of related genome areas and obtain some clue of transcriptional activity. Taken together, this study could provide a new reliable foundation for research on the Chinese medaka, as well as for its use as a model organism.

## Methods

### *De novo* genome assembly

The samples of *O. sinensis* were obtained from the National Plateau Wetland Research Center, Southwest Forestry University; muscle tissue was used for nucleic acid extraction. High-quality purified RNA was used to construct a transcript sequencing library and DNA was used to construct stLFR, Nanopore long-reads, Hi-C sequencing, and ATAC-seq libraries. stLFR technology added the same barcode sequence to subfragments of the original long DNA molecule, allowing these co-barcoded sub-fragments to be subsequently sequenced using a second-generation platform^6^. This method generates long reads with high accuracy, enabling high-quality assembly and subsequent analysis. Most importantly, stLFR was cost-effective and has been widely used in aquatic genome projects^7,8^.

The genome size and heterozygosity of *O. sinensis* were estimated using Jellyfish v2.2.6^9^ and GenomeScope v1.0.0^10^ by k-mer analysis with clean stLFR data. The clean data was then used in *de novo* genome assembly with the stlfr2supernova pipeline (https://github.com/BGI-Qingdao/stlfr2supernova_pipeline). This pipeline conducts *de novo* assembly using stLFR data with Supernova Assembler, which refers to the de novo software from 10X Genomics^11^. Nanopore long-reads were employed to carry out further scaffolding, gap-closing, and polishing at the same time using TGS-GapCloser^12^. The size of the assembly after these steps was bigger than the k-mer result (approximately 906 Mb) so purge_haplotigs^13^ was applied to remove redundancy. Finally, a draft assembly that covered approximately 796 Mb of the genome with a contig N50 length of 373.71 Kb was obtained.

### Hi-C analysis and chromosome assembly

**Fig. 1:**
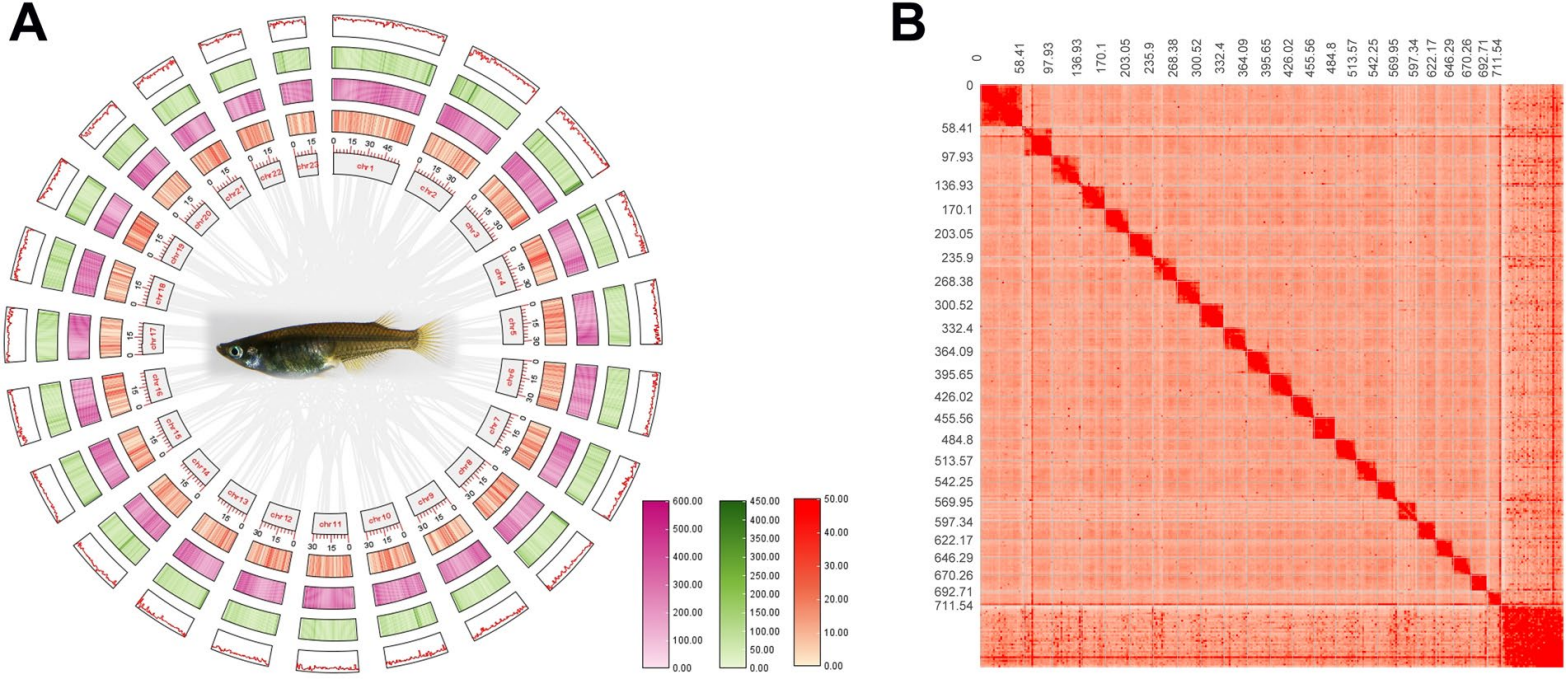
(**A**) Global genome landscape of Chinese medaka. From inner to outer circles: Density of genes with 500 kbp windows, ranging from 0 to 50; Density of TE with 500 kbp windows, ranging from 0 to 600; Density of TRF with 500 kbp windows, ranging from 0 to 450; Distributions of GC content with 500 kbp windows. (**B**) Hi-C interaction heat map and scatter plot of genome assembly GC content and sequencing depth.

The Hi-C reads were aligned to the draft assembly using HiCPro^14^ to find valid read pairs. Juicer^15^ and 3D-DNA^16^ were then used to finish the construction of chromosomes, with manual correction of misjoins, wrong order, and opposite orientation using Juicebox^15^. The Hi-C scaffolding resulted in 23 pseudo-chromosomes with a total length of 712.17 Mb. We offer a chromosome circus map using TBtools^17^ and heatmap using Juicebox^18^ (Fig. 1A,B). Genome assembly statistics are shown in Table 1 and Table 2. BUSCO (Benchmarking Universal Single-Copy Orthologs)^19^ evaluation of the assembly reached 95.1% using actinopterygii_odb9 database, indicating a well-covered fish genome assembly (Table 3).

### Repeat annotation

Tandem repeats and interspersed repeats identification was essential before protein-coding gene and function annotations. RepeatMasker^20^ and RepeatProteinMask were applied to find repeat elements as homology predictions based on RepBase^21^. For the *de-novo* method, RepeatModeler^22^ was used to predict repeat elements; LTR_FINDER^23^, and TRF tool^24^ were also used to predict repeat elements based on sequences features. Overall, 311.32 Mb of the *O. sinensis* genome assembly were identified as repetitive elements, accounting for 39.05% of the whole genome (Table 4).

**Table 4:** Prediction of repeat elements.

Type	Repeat Size(bp)	% of genome
**TRF**	12,455,455	1.56
**RepeatMasker**	104,455,645	13.10
**RepeatProteinMask**	31,952,649	4.01
***De novo***	293,230,747	36.78
**Total**	311,329,140	39.05

### Gene prediction and annotation

Protein coding genes were predicted with multiple sources of evidence including homology-based alignments, *de novo* prediction, and transcriptome-assisted methods. In homology alignments, the protein sequences of *Danio rerio* (GRCz11), *Ictalurus punctatus* (IpCoco_1.2), *Oryzias javanicus* (OJAV_1.1), *Oryzias latipes* (ASM223467v1), *Oryzias melastigma* (Om_v0.7), *Poecilia formosa* (PoeFor_5.1.2), and *Takifugu rubripes* (FUGU5) were mapped to a repeat soft masked draft genome using blat^25^, and Genewise was applied to define gene models^26^. In *de-novo* prediction, Augustus^27^ was used to predict the coding regions of genes. In transcriptome-assisted methods, two different methods were used to obtain predicted gene sets. First, RNA reads were mapped to the genome assembly using Hi-SAT2^28^; the result was used to build a transcript gene model using Stringtie^29^ and TransDecoder. Second, a transcriptome was first assembled with RNA reads using Trinity, before PASA was used to get a gene model. Finally, all evidence was merged to form a consensus gene structure annotation result using GLEAN^30^. A total of 22,461 protein-coding genes were successfully predicted. BUSCO^19^ assessments reached 94.6% using the actinopterygii_odb9 database, indicating relatively complete gene annotation coverage (Table 3).

All predicted genes were compared with public biological function databases including KEGG^31^, Swissprot^32^, and TrEMBL^33^ (https://www.uniprot.org/statistics/TrEMBL) using BLASTp^34^ with an E-value cutoff of 1e-5 for functional annotation. Interpro was applied to provide functional analysis of protein sequences by classifying them into families and predicting the presence of domains and important sites, followed by Gene Ontology annotation^35–37^. Overall, 22,162 protein-coding genes (98.67%) were successfully functionally annotated (Table 5).

**Table 5:** Genome function annotation result.

	Number	Percentage (%)
Total	22,461	100
Swissprot-Annotated	20,906	93.08
KEGG-Annotated	19,352	86.16
TrEMBL-Annotated	22,135	98.55
Interpro-Annotated	20,291	90.34
Overall	22,162	98.67

Non-coding RNAs (ncRNAs) are an important part of genome annotation as they can be active in transcriptional and translational regulation of gene expression as well as in the modulation of protein function^38^. Since ribosomal RNA (rRNA) was highly conservative, vertebrate rRNA data was used as a reference to map to the draft genome using BLASTn with an E-value of 1e-5. For the discovery of transfer RNA (tRNA), tRNAscan-SE v1.3.1 was applied with eukaryotic parameters according to the characteristics of tRNA^39^. Rfam database was applied to find microRNAs (miRNA) and small nuclear RNA (snRNA)^40,41^. In total, 676 rRNA, 740 tRNA, 231 miRNA, and 1,168 snRNA genes were identified from the *O. sinensis* genome.

### Chromosome accessibility analysis using ATAC-seq

**Fig. 2:**
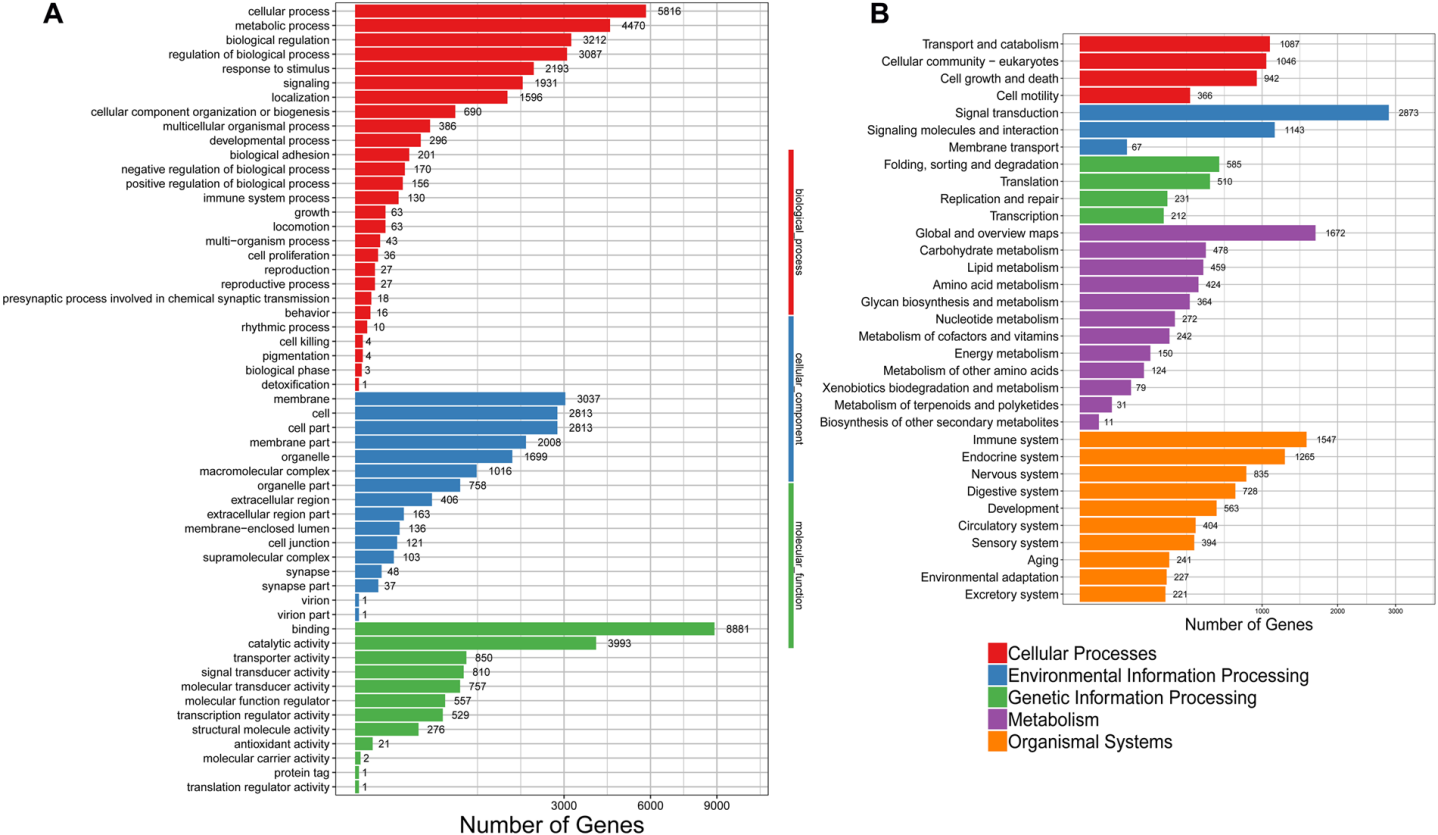
ATAC-seq peaks result from related genes’ functional enrichment. (**A**) GO enrichment; (**B**) KEGG enrichment.

ATAC-seq is a widely used method used to identify regions of open chromatin in genomes. SOAPnuke (v1.5.2)^42^ was used to filter out reads with low quality original data or high content of unknown bases. Bowtie2 (v2.2.5)^43^ was used to align reads to the *O. sinensis* genome assembly and peak calling was performed with MACS2 software (v2.1.0)^44^. Peaks related to genes were used to carry out GO and KEGG enrichment analysis (Fig. 2).

## Data Records

The Nanopore long reads, stLFR genomic sequencing data, Hi-C data as well as the assembled genome have been deposited at China National GeneBank DataBase (CNGBdb) under the accession CNP0003475^45^^.^The whole sequencing dataset of *O. sinensis* was deposited in the NCBI Sequence Read Archive database (https://www.ncbi.nlm.nih.gov/bioproject/PRJNA895195) under project identification number PRJNA895195. This Whole Genome Shotgun project has been deposited at DDBJ/ENA/GenBank under the accession JAUDJI000000000. The version described in this paper is version JAUDJI010000000^46^. Sequence Read Archive (SRA) project number was SRP410304^47^. DNA sequencing data from the WGS library were deposited in the SRA at SRR22435960^48^. DNA sequencing data from the Hi-C library were deposited in the SRA at SRR22435961^49^. The ATAC-sequencing data were also deposited at Figshare^50^^.^

## Technical Validation

To evaluate the quality of the genome assembly, stLFR reads were mapped to the final reference genome assembly using BWA (v0.7.12). A total of 98.27% of reads were mapped, covering 98.29% of the genome sequence. Genome average sequencing depth reached 172x, and 97.34% of the genome had a sequencing depth of over 20x. The scatter plot between assembly sequencing depth and GC content found no abnormal GC content, recognized as no exogenous pollution. The completeness of the genome assembly and annotation was assessed using BUSCO (v3.0) with the actinopterygii_odb9 database. The BUSCO benchmark of genome assembly and gene annotation reached 95.1% and 94.6%, respectively.

## Data Availability

All software used in this study are in the public domain, with parameters described in Methods and this section. If no detailed parameters were mentioned for the software, default parameters were used according to the software introduction.
